# Easy domain adaptation method for filling the species gap in deep learning-based fruit detection

**DOI:** 10.1038/s41438-021-00553-8

**Published:** 2021-06-01

**Authors:** Wenli Zhang, Kaizhen Chen, Jiaqi Wang, Yun Shi, Wei Guo

**Affiliations:** 1 Information Department, Beijing University of Technology, Beijing, 100022 China; 2 Institute of Agricultural Resources and Regional Planning, Chinese Academy of Agricultural Sciences, Beijing, 100081 China; 3 International Field Phenomics Research Laboratory, Institute for Sustainable Agro-Ecosystem Services, Graduate School of Agricultural and Life Sciences, The University of Tokyo, Tokyo, 188-0002 Japan

**Keywords:** Bioinformatics, Field trials

## Abstract

Fruit detection and counting are essential tasks for horticulture research. With computer vision technology development, fruit detection techniques based on deep learning have been widely used in modern orchards. However, most deep learning-based fruit detection models are generated based on fully supervised approaches, which means a model trained with one domain species may not be transferred to another. There is always a need to recreate and label the relevant training dataset, but such a procedure is time-consuming and labor-intensive. This paper proposed a domain adaptation method that can transfer an existing model trained from one domain to a new domain without extra manual labeling. The method includes three main steps: transform the source fruit image (with labeled information) into the target fruit image (without labeled information) through the CycleGAN network; Automatically label the target fruit image by a pseudo-label process; Improve the labeling accuracy by a pseudo-label self-learning approach. Use a labeled orange image dataset as the source domain, unlabeled apple and tomato image dataset as the target domain, the performance of the proposed method from the perspective of fruit detection has been evaluated. Without manual labeling for target domain image, the mean average precision reached 87.5% for apple detection and 76.9% for tomato detection, which shows that the proposed method can potentially fill the species gap in deep learning-based fruit detection.

## Introduction

There is a vital need in the horticulture research field to understand fruit-related phenotypic traits, such as fruit number, size, and color. With the rapid development of modern computer technology, the demand for visual detection techniques in agriculture has increased. An object detection technique can obtain the location and category information of the fruit in the image, such as fruit positioning^1,2^, fruit estimation^3,4^, and automatic fruit picking^5,6^, which is the technical basis for intelligent work in the orchard.

Recently, owing to the advantages of deep learning-based object detection techniques^7–13^, which perform high detection accuracy and good model robustness, they have gradually replaced traditional detection methods and are widely applied in orchard fruit detection. On the other hand, most deep learning-based fruit detection techniques adopt the supervised learning strategy, which requires a large number of labeled fruit image datasets to train the model. However, a model generated with a dataset collected for one species may not work for another species; hence, new species always require labeling new data to train the new model, which is labor-intensive and time-consuming. Therefore, reducing the dataset labeling workload has become a topic of intense interest^14^.

In the current stage, most related works use a strongly supervised labeling method^15^ that requires drawing bounding boxes around the target objects with location and category information for model training. Mu et al.^16^ collected fruit images of tomatoes in a greenhouse, Wang et al.^4^ collected mango fruit images at night orchards, then labeled each visible target fruit in the images by tight bounding boxes manually. Although the strongly supervised labeling method provides better detection performance, the labeling cost were high and time-consuming.

Some works then tried to train detection models based on weakly-supervised labeling methods to reduce the labeling cost. For example, researchers used image-level labels^17–20^ (providing information on the category of objects in the image, no specific location information) and dot labels^21^ (marking object location information with dots) to reduce the overall cost and time consumption by lessening the labeling time of individual labels. Bellocchio et al.^22,23^ proposed a weakly supervised deep architecture that relies only on an image level binary classifier(whether the image contains instances of the fruit or not) to train the fruit counting model on source images. The unsupervised transformation learning and pseudo-label process are further combined to generate target fruit images and related labels and then applied to the fruit counting task on target images. Because pseudo labels are acquired only for the generated fruit images and different from actual target fruit images, the model did not fit well with the actual target fruit images. Lu et al.^24^ used dot annotated method to perform maize tassel counting task in localized regions of the farmland. Ghosal et al.^25^ proposed active learning inspired weakly supervised deep learning framework, and Lagandula et al.^26^ combined dot-annotated methods with active learning methods^27,28^ to reduce labeling time cost more than 50% on sorghum and wheat images. However, the weakly supervised labeling method still requires a certain amount of manual data labeling work.

Some researchers also suggested that unsupervised learning methods^29–31^ can be applied to agriculture since they do not require data labeling. Wachs et al.^29^ proposed a method based on K-means clustering to achieve the unsupervised detection of green apples in infrared and RGB images with an accuracy of 53.2%. Dubey et al.^30^ utilized the K-means clustering algorithm to perform fruit segmentation and localization based on color features. Zhang et al.^31^ proposed an unsupervised learning conditional random field image segmentation algorithm to segment plant organs such as fruits, leaves, and stems from green house plant images without manual labeling. However, in most agricultural field work, because of the complexity of the context and the diversity of objectives in the actual scenario, unsupervised learning methods did not performed as accurate as supervised learning methods. To address the high dataset labeling cost, some researchers also suggest that public available datasets^32–37^ can be used to train fruit detection models. Sa et al.^32^ presented the DeepFruit dataset, which contains apple, avocado, capsicum, mango, orange, rockmelon, and strawberry; Bargoti et al.^33^ presented a acfr-multifruit-2016 dataset that contains mango, almond, and apple; Muresan et al.^34^ presented Fruit-360 dataset that contains 131 categories of fruit images with a single background. However, owing to the different image acquisition conditions in each fruit dataset, including lighting conditions, occluding conditions, and shooting distance, the trained fruit detection model showed low generalization ability when applied to real applications, and it is also kown that train a model based on target scenes will always performs best.

Therefore, we consider to train several locally good models for each domain based on their own data for fruit detection tasks. Then the main problem shifts to how to generate labeled data for new domain efficiently, which the Generative Adversarial Networks (GAN)^38^ seems to be a powerful tool for it. GAN have been widely used for image transformation tasks. Stein et al.^39^ and Zhang et al.^40^ proposed a GAN-based image transformation method to implement image transformation between simulated and real images for cross-domain segmentation tasks. Roy et al.^41^ proposed Semantic-Aware GAN, which introduces multiple loss functions to optimize model training and can be applied to image transformation between image domains with large geometric shape differences. Valerio et al.^42^ proposed to combine multiple regression leaf counting model and adversarial network idea to achieved cross-domain leaf counting for in the unlabeled target domain by extracting domain invariant features from different plant species. However, the above research mainly focuses on improving the generated image quality for image transformation, not labeling images for the new target domain. So in this paper, we propose a new method to use GAN to automatically label different fruit image datasets by only using a set of existing labeled fruit images.

The proposed method first uses the CycleGAN^43^ network to transfer the source domain fruit dataset (with labeled information) to the target domain fruit dataset (without labeled information), then applies the pseudo-label method to label the target fruit dataset. Finally, it uses a self-learning method of pseudo labels further to improve the labeling accuracy. The performance of the proposed method from the perspective of fruit detection has been evaluated then by a labeled orange image dataset and unlabeled apple and tomato image dataset.

## Materials and methods

### Dataset acquisition

The experiments in this paper contain two datasets: CycleGAN datasets and object detection datasets.

#### CycleGAN datasets

The image transformation experiments used the apple2orange dataset^43^ and the orange2tomato dataset.

(1)The apple2orange dataset contains orange and apple to train the image transformation model between orange and apple. The training set containes 995 apple images and 1019 orange images, while the test set containes 266 apple images and 248 orange images, with a uniform image resolution of 256 × 256 pixels.

(2)The orange2tomato dataset contains orange images from apple2orange dataset and the tomato images collected from the Internet. The training set contains 654 tomato images and 1019 orange images, while the test set contains 102 tomato images and 248 orange images, with a uniform image resolution of 256 × 256 pixels.

#### Object detection datasets

The following source fruit dataset and target fruit dataset were used in the fruit detection experiments:

(1) **Source orange dataset:** The dataset was collected from an orange orchard in Sichuan Province, China. In total, 664 orange images were collected using a DJI Osmo Action camera (Shenzhen DJI Science & Technology Co., Ltd.), including down-light, back-light, dense target, blocking target, and other fruit scenes. Relevant annotation tools were exploited to obtain the coordinate information of each orange annotation box, i.e., the *x* and *y* coordinates of the two points in the upper left and lower right corners of the annotation box. Afterward, the images were resized to 416 × 416, and randomly divided into a training set and a test set according to a 7:3 ratio.

(2) **Target dataset: apple and tomato dataset:**

Target apple dataset: The dataset is based on the MineApple dataset^37^, which contains images of red and green apples in a variety of highly cluttered environments, with an average target fruit size of 40 × 40 pixels. In total, 504 images of red apples from the original training set were selected as the experimental training set, with an image resolution of 1280 × 720 and no data labeling. In total, 82 red apple images from the original test set were selected as the experimental test set. The images were cropped to 719 × 898 to remove the influence of fallen apples on the ground and then been labeled with relevant labeling tools for later experimental validation.

#### Target tomato dataset

The dataset is based on the dataset published by Mu et al.^16^, which ware collected from two farms in Tokyo, Japan. The collected tomato images were pre-processed and the image resolution was set to 1920 × 1080, where the training set consisted of 598 unlabeled tomato images and the test set consisted of 150 labeled tomato images.

Among them, the orange images and apple images were collected outdoors, and the tomato images were collected indoors. Besides, most of the tomato images includes green tomato fruits, so the color features are similar to the background leaves. The differences in these collection environments, locations and shooting distances bring significant challenges to this study.

### Workflow of the proposed method

**Fig. 1:**
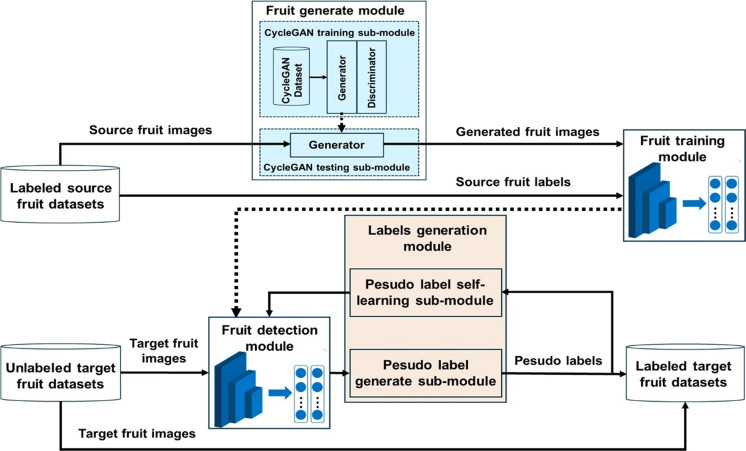
Workflow of data labeling conversion method. The method mainly includes fruit generate module, fruit training/detection module and label generation module, to relize the automatic data labeling of unlabeled fruit datasets

In this paper, a data labeling conversion method between different species of fruits is proposed to realize the automatic data labeling of unlabeled fruit datasets and save the dataset labeling cost in detection tasks. The flowchart of the algorithm is depicted in Fig. 1.

The application context comprises a labeled source fruit dataset *D*_*s*_ and an unlabeled target fruit dataset $D_T^U$, both from Object detection dataset. We assume the sets $D_s = \{ \left( {I_S^1,l_S^1} \right),\left( {I_S^2,l_S^2} \right), \ldots \ldots ,\left( {I_S^{N_S},l_S^{N_S}} \right)\}$ and $D_T^U = \{ I_T^1,I_T^2, \ldots \ldots ,I_T^{N_T}\}$, where *I*_*S*_ and *I*_*T*_ represent the image in the source fruit dataset and the target fruit dataset, respectively. *l*_*S*_ represents the labeling information of the corresponding images in the source fruit dataset, and *N* represents the number of images in the dataset. The overall steps of the method are as follows:

**Step 1**: The fruit images were imported from the dataset *D*_*s*_ into the CycleGAN testing network for image transformation (the CycleGAN network is noted as *M*_*1*_ and the associated model weight parameter is noted as *w*_*1*_); there upon, construct a fake apple dataset *D*_*F*_ with the labeling information of the source fruit dataset *D*_*s*_, where $D_F = \left\{ {\left( {I_F^1,l_S^1} \right),\left( {I_F^2,l_S^2} \right), \ldots \ldots ,\left( {I_F^{N_S},l_S^{N_S}} \right)} \right\}$ and *I*_*F*_ represents the transformed fake target fruit image.

**Step 2**: Feed dataset *D*_*F*_ into the fruit detection model called Improved-Yolov3^37^ for training, the obtained fruit detection model is noted as *M*_*2*_, and the weight parameter of the model is noted as *w*_2_.

**Step 3**: Using the dataset $D_T^U$ as the test set input model *M*_*2*_, obtain the detection box of the real target fruit in the image *I*_*T*_, and treat the detection box as the pseudo-label information of the image *I*_*T*_. Subsequently, use the self-learning method of the pseudo label to improve the accuracy of the labels. Finally, obtain the dataset $D_T^U$ with pseudo labels and note as $D_T^L$, where $D_T^L = \{ (I_T^1,l_T^1),(I_T^2,l_T^2), \ldots \ldots ,(I_T^{N_T},l_T^{N_T})\}$ and *l*_*T*_ represent the labeling information for the associated image *I*_*T*_.

**Step 4**: Output the above dataset $D_T^L$ with label information.

The data labeling conversion algorithm includes the implementation of the following four functional modules.

#### A: Image transformation

The generative adversarial network^38^ has been one of the most popular models in recent years. The model mainly improves the performance of the discriminator network in distinguishing true and false images and guides the generator network to output more realistic images through the zero-sum game between the generator network and the discriminator network. In this study, the CycleGAN^43^ network was deployed to realize image transformation among different species of fruits.

**Fig. 2:**
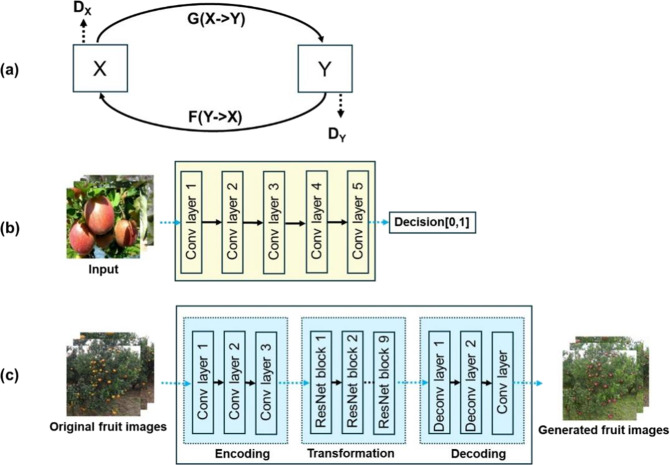
Image transformation network related components. **a** represents a mapping function diagram between two image domains X and Y, including two mappings, G:X->Y and F:Y->X, and two discriminators, ***D***_***X***_ and ***D***_***Y***_; **b** represents the discriminator network diagram; and **c** represents the generator network diagram

The purpose of the CycleGAN network is to learn the domain mapping between two image domains, X (source domain) and Y (target domain), through unpaired sample images in the dataset, thereby realizing the image transformation between domains without supervision. As shown in Fig. 2a, the CycleGAN network includes two generator networks G and F, for image transformation between two image domains in different directions, and two discriminator networks *D*_*X*_ and *D*_*Y*_. The generator network (Fig. 2c) consists of an encoder, a transformer, and a decoder, which operate as follows: first, the source domain image is input into the encoder and the image feature vector is extracted. Afterward, the source domain feature vector is transformed into a target domain feature vector by a transformer, which consists of a residual module constructed of two convolutional layers; this enables the retention of the feature information in the image of the source domain while transforming. Finally, the feature vector of the target domain image output from the transformer is passed through the deconvolution network to reconstruct the low-level features and generate the target domain image. In addition, the discriminator network (Fig. 2b) mainly consists of convolutional layers, which are firstly used to extract image features. The extracted feature vectors are thereupon determined by the one-dimensional output convolutional layer of the last layer and the authenticity of the image is finally determined.

**Fig. 3:**
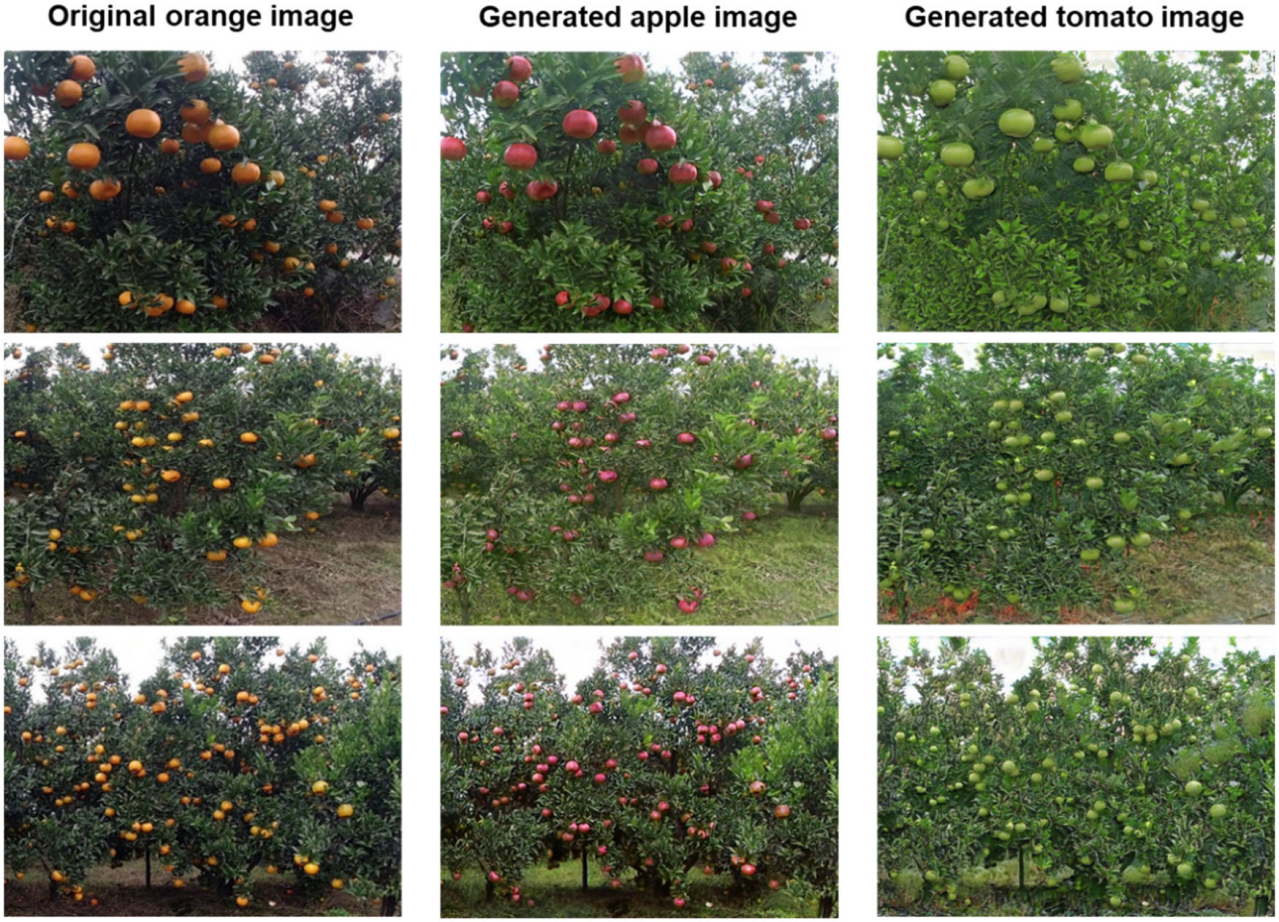
Examples of source fruit image and generated fake fruit image. The figure shows the image transformation effects at different shooting distances, where the first column shows the source orange image, and the second column shows the generated fake apple image and the third column shows the generated fake tomato image

To address the problem of large differences in features between fruits of different species, this paper implements feature transfer between fruits, which is more effective in allowing the model to learn the target fruit features directly. When the CycleGAN network training is completed, the generator network can be used to realize image transformation for different species of fruit images. The operation is as follows. First, train CycleGAN network using different species of source fruit dataset and target fruit dataset, both from CycleGAN dataset, and the image input size of the CycleGAN network is 256*256. Second, using the trained CycleGAN network, according to Eq. (1), transform the source fruit image $I_S^i$ in the dataset *D*_*s*_ into the fake target fruit image $I_F^i$ (as shown in Fig. 3), where *w*_1_ represents the weight parameter of the CycleGAN network. By combining the original labeling information in the dataset *D*_*s*_, the fake fruit dataset *D*_*F*_ with the source fruit labeling information was constructed.

1 $$I_F^i = M_1\left( {w_1,I_S^i} \right),i = 0,1,2,......,N_S$$

Finally, obtain the fruit detection model *M*_2_ by training dataset *D*_*F*_, which could be applied to the detection task of the dataset $D_T^U$.

#### B: Fruit detection network

**Fig. 4:**
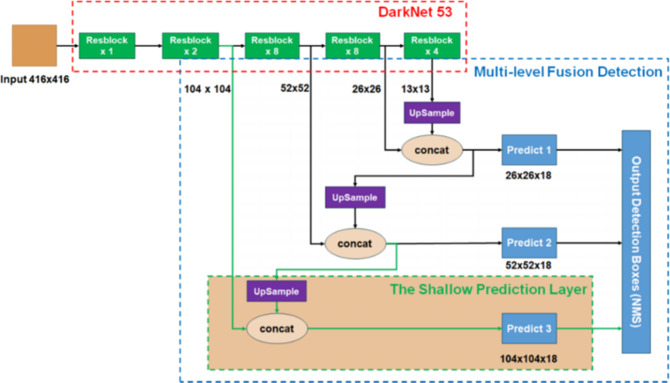
Structure of the Improved-Yolov3 model. The model mainly consists of Darknet53 backbone, FPN network structure and multi-level detection head

The detection model applied in this study is grounded on Improved-Yolov3^44^. The model structure is depicted in Fig. 4. Improved-Yolov3 is designed based on the original Yolov3 model, which removes the deep network detection branch with a downsampling rate of 32 and adds a shallow network detection branch with a downsampling rate of 4, fuse the deep and shallow network features by Feature Pyramid Network(FPN) network structure, to improve the small-scale fruit detection performance. More detailed information on Improved-Yolov3 can be found at^44^.

#### C: Pseudo-label generation

The traditional dataset label is based on manual labeling, while pseudo labeling is a machine-generated bounding box similar to manual labeling. This paper proposes a pseudo-labeling approach to generate labels in the dataset $D_T^U$ automatically. Because the fruit features of the fake fruit images generated by the CycleGAN network are more similar to those of naturally grown target fruit images, the model *M*_2_ has some ability to detect real target fruits. Therefore, the labeling information (pseudo label) in the dataset $D_T^U$ can be obtained by the model *M*_2_. The operation is as follows.

First, use the fruit detection model *M*_2_ to obtain the detection bounding box information for real target fruit images in the dataset $D_T^U$. Thereupon, utilize the acquired detection bounding box as pseudo label of the dataset $D_T^U$ to construct the dataset $D_T^L$ with labeling information automatically and realize the conversion of labeling information between different species of fruit datasets.

#### D: Pseudo-label self-learning

The detection bounding box obtained by the model *M*_2_ in real target fruit images *I*_*T*_ is used as a pseudo label, and because the model *M*_2_ is trained from the fake fruit dataset *D*_F_, it is prone to the presence of a false detection bounding box in real target fruit images *I*_T_, resulting in noise in the generated pseudo label. Therefore, how to reduce the impact of noise in pseudo labels is one of the main research points in this paper.

In the process of acquiring pseudo labels, the setting of the confidence threshold is related to the quality and quantity of the acquired pseudo labels. When the confidence threshold higher, the acquired pseudo label has a higher probability of correctly labeling the target fruit in the image, while a high confidence threshold leads to a lower number of pseudo labels, and the opposite is also true. Therefore, this paper proposes a pseudo-label self-learning method, which includes a pseudo-label noise filtering operation and a cyclic update operation to reduce the effects of pseudo-label noise, thereby improving the labeling accuracy of pseudo labels, as shown in Algorithm 1. The pseudo-label self-learning method is described as follows.

Pseudo-label noise filtering: First, set the initial confidence threshold *θ* . The unlabeled target fruit dataset $D_T^U$ is used as the test set input model *M*_2_ to obtain all the detection boxes, as shown in the following equation.

2 $$\mathop {\sum}\limits_{j = 0}^{N_i - 1} {l_T^{ij}} = M_2(w_2,I_T^i,\theta )$$

where $l_T^{ij}$ denotes the *j*th detection box information of the i^th^ real target fruit image and *N*_*i*_ denotes the total number of detection boxes for the i^th^ real target fruit image, where $i = 0,1,2,......,N_T$−1. Subsequently, count the sum of the scores of all detection boxes and calculate the average score *S*_*aver*_ according to Eq. (3), filter out the detection boxes below the average score *S*_*aver*_, and the higher score of the detection box is regarded as the pseudo label of the real target fruit dataset $D_T^U$, as shown in Eq. (4).

3 $$S_{aver} = \frac{{Score(\mathop {\sum }\nolimits_{i = 0}^{N_T - 1} \mathop {\sum }\nolimits_{j = 0}^{N_i - 1} l_T^{ij})}}{{\mathop {\sum }\nolimits_{i = 0}^{N_T - 1} N_i}}$$

4 $$\mathop {\sum}\limits_{j = 0}^{N_i - 1} {l_T^{ij}} = Filter(\mathop {\sum}\limits_{j = 0}^{N_i - 1} {l_T^{ij}} ,S_{aver})$$

where the *Score* function indicates that the scores of the acquired detection boxes are summed and the *Filter* function indicates that the detection boxes below the set score value are filtered.

Pseudo-label cycle update: When the model *M*_2_ is fine-tuned using the real target fruit dataset $D_T^L$ for a certain number of epochs, the model *M*_2_ learns the features of the real target fruit image, improves the detection performance of the real target fruit image. At this time, the detection box of the unlabeled real target fruit dataset $D_T^U$ obtained by the model *M*_2_ is more comprehensive and accurate, and the labeling accuracy of the pseudo label is higher. Therefore, the method in this study re-obtains the detection box of the dataset $D_T^U$ by using the current fruit detection model *M*_2_ at certain intervals of training epochs. The pseudo-label information of the unlabeled dataset $D_T^U$ is updated by the aforementioned pseudo-label noise filtering method to improve the labeling accuracy.

**Table:** 

**Algorithm 1: Pseudo-label Self-learning**
**Input** Unlabeled Images *I*_*T*_, labeled dataset *D*_*F*_, Object Detector *M*_2_, Confidence threshold *θ*, Number of pseudo-label updates N **Output** Label *l*_*T*_ 1: Initialize *M*_2_ with *D*_*F*_ 2: for *n* ← 1 to N do: 3: Input *M*_2_ with *I*_*T*_, obtain *l*_*T*_ 4: Filter noise label *l*_*T*_ based on Eqs. (2)–(4), obtain labeled dataset *D*_*T*_ 5: Update *M*_2_ via fine-tuning with *D*_*T*_ 6: **end** 7: **Output**: label *l*_*T*_

### Experimental setup

This experiment deploys a deep learning framework for model training and testing on a computer platform with an Intel Core i7-8700K CPU processor (32GB of RAM), GeForce GTX 1080Ti GPU graphics card (12GB of video memory), and an operating system with ubuntu18.04LTS, using the Python 3.6.5 programming language to implement the construction, training, and validation of network models under the Pytorch 1.0.0 deep learning framework.

CycleGAN model training: The network was trained using a mini-batch adaptive moment estimation (Adam) optimizer with a momentum factor of 0.5 and a batch size of one. The learning rate for the first 100 training epochs was set to 0.0002, the learning rate for the next 100 training epochs was set to zero with linear recession, and other relevant parameter information from the original paper^43^ was applied.

Improved-Yolov3 model training: The detection model is trained in a computer hardware environment with a GPU to improve the convergence rate of model training. Stochastic gradient descent with a mini-batch with a momentum factor was used to train the network. The value of the momentum factor was set to 0.9, the decay was 0.0005, and the batch size was four, the initial learning rate was 0.001, and the learning rate was adjusted using the cosine annealing function. A larger learning rate in the early stage helps the network converge quickly, and a smaller learning rate in the later stage made the network more stable and obtains the optimal solution.

### Evaluate metrics

To evaluate the detection performance of the Improved-Yolov3 model, this paper uses Precision, Recall, F1 score, and mAP as the evaluation metrics. A predicted bounding box is considered correct (true positive) if it overlaps more than the intersection-over-union threshold with a labeled bounding box. Otherwise, the predicted bounding box is considered false positive. When the labeled bounding box has an intersection over union with a predicted bounding box lower than the threshold value, it is considered false negative. The standard intersection-over-union threshold value of 0.5 was adopted. The relevant formulae are shown in the following equations.

5 $$Precision = \frac{{Tp}}{{Tp + Fp}} \times 100{\mathrm{\% }}$$

6 $$Recall = \frac{{Tp}}{{Tp + Fn}} \times 100{\mathrm{\% }}$$

7 $$F1\,Score = \frac{{2 \times Precison \times Recall}}{{Precison + Recall}} \times 100{\mathrm{\% }}$$

8 $$mAP = J(Precison,\,Recall)$$

where *J*(•) represents the area calculation function under Precision and Recall curves.

## Results

The datasets used in this experiment are described below:

(1)Dataset *D*_*S*_: contains the images of source oranges and the associated labeling information.

(2)Dataset $D_{T\_apple}^U$: contains the images of real apples without labeling information.

(3)Dataset $D_{T\_{\mathrm{tomato}}}^U$: contains the images of real tomatoes without labeling information.

### Evaluation of datasets *D*_*S*_ and *D*_*F*_

In this study, the fruit detection model Improved-Yolov3^44^ was trained and tested using the dataset *D*_*S*_ and the dataset *D*_*F*_, respectively. *D*_*S*_ contains source orange dataset *D*_*S_*orange_, and *D*_*F*_ contains fake apple datasets *D*_*F_*apple_ and fake tomato datasets *D*_*F_tomato*_. As shown in Table 1, the mAP value obtained by the model Improved-Yolov3 tested in the dataset *D*_*S_*orange_ is 95.1%. Because the fake apple image and the fake tomato image were obtained by transforming the orange fruit image in the dataset *D*_*S*_, the fruit location information is the same in both datasets, with the main divergence being that the underlying features in the image, such as fruit color and texture, are different. After testing, the mAP value of the Improved-Yolov3 model on the dataset *D*_*F_*apple_ and *D*_*F_tomato*_ are 94.8% and 96.7%, respectively; hence, the difference between the values of each experimental metric on the datasets *D*_*S*_ and *D*_*F*_ is not large, and both have high detection accuracy.

**Table 1:** Evaluation results for datasets *D*_*S*_ and *D*_*F*_ in the Improved-Yolov3 model

Model	*Datasets*	Precision	Recall	F1 Score	mAP
Improved-Yolov3	*D*_*S_orange*_	0.886	0.923	0.904	0.951
Improved-Yolov3	*D*_*F_apple*_	0.889	0.920	0.904	0.948
Improved-Yolov3	*D*_*F_tomato*_	0.913	0.941	0.927	0.967

### Attachment

The following is the attachment related to this paper, mainly including the picture form of the related table.

### Adding pseudo labels obtained through different confidence thresholds

As shown in Tables 2, 3, for models obtained from pseudo labels that fine-tune at different confidence thresholds, this experiment was conducted to compare the test results of real apple images and real tomato images. Because there are certain differences in the features between the fake fruit images generated by the CycleGAN network and the natural real-grown fruit images, the model *M*_2_ is fine-tuned using a pseudo-labeling method to reduce the learned feature variability by fitting the feature distribution of the real fruit images. The experiments in this study obtain pseudo labels for the dataset $D_{T\_{\mathrm{apple}}}^U$ and $D_{T\_tomato}^U$ by setting different confidence thresholds, and the quality and quantity of pseudo labels varied depending on the confidence threshold settings, which impacted fruit detection model *M*_2_. The confidence threshold values ranged from 0.1 to 0.9, and the interval between the values under experimental comparison was 0.1. (The bolded part of the following table indicates the model performance results obtained under the current optimal confidence threshold parameters).

**Table 2:** Label conversion of orange dataset to apple dataset: the pseudo-labeling method obtaining pseudo labels by setting different confidence thresholds, generating a real apple dataset ${\boldsymbol{D}}_{{\boldsymbol{T}}\_{\boldsymbol{apple}}}^{\mathbf{L}}$ with labeling information, and finally verifying the validity of the generated labels by the model’s detection performance

Model	Pseudo label	Conf	Precision	Recall	F1 Score	mAP
Improved-Yolov3	×	None	0.704	0.658	0.68	0.653
	**√**	0.1	0.724	0.72	0.722	0.769
	**√**	0.2	0.747	0.746	0.746	0.788
	**√**	0.3	0.768	0.769	0.768	0.805
	**√**	0.4	0.783	0.786	0.784	0.828
	**√**	0.5	0.79	0.8	0.795	0.829
	**√**	**0.6**	**0.803**	**0.808**	**0.805**	**0.852**
	**√**	0.7	0.813	0.822	0.817	0.845
	**√**	0.8	0.815	0.798	0.806	0.843
	**√**	0.9	0.79	0.796	0.793	0.836

**Table 3:** Label conversion of orange dataset to tomato dataset: the pseudo-labeling method obtaining pseudo labels by setting different confidence thresholds, generating a real tomato dataset ${\boldsymbol{D}}_{{\boldsymbol{T}}\_{\boldsymbol{tomato}}}^{\mathbf{L}}$ with labeling information, and finally verifying the validity of the generated labels by the model detection performance

Model	Pseudo label	Conf	Precision	Recall	F1 Score	mAP
Improved-Yolov3	×	None	0.723	0.725	0.724	0.711
	**√**	0.1	0.753	0.751	0.752	0.729
	**√**	0.2	0.754	0.756	0.755	0.732
	**√**	0.3	0.745	0.747	0.746	0.738
	**√**	0.4	0.769	0.767	0.768	0.741
	**√**	**0.5**	**0.77**	**0.769**	**0.769**	**0.752**
	**√**	0.6	0.765	0.765	0.765	0.748
	**√**	0.7	0.76	0.759	0.759	0.745
	**√**	0.8	0.748	0.747	0.748	0.744
	**√**	0.9	0.705	0.708	0.707	0.688

When the real fruit image is tested directly using the model *M*_2_ obtained from the dataset *D*_*F*_, the mAP value obtained from the real apple and tomato datasets were 65.3% and 71.1%. When using the pseudo-labeling method, as the set confidence threshold increased, the accuracy of the pseudo-label labeling increases, the noise in the pseudo label decreases, and the mAP of the model tends to increase incrementally. When the confidence threshold exceeds a certain value, the mAP value of the model at that time decreases as the confidence threshold value increases, and the reason for the analysis is that the low number of pseudo label with high threshold leads to a decrease in the diversity of features learned, which affects the generalization ability of the model. The model mAP value reached 85.2% when the confidence threshold was 0.6 in the real apple dataset (as shown in Table 2). The model mAP value reached 75.2% when the confidence threshold was 0.5 (as shown in Table 3) in the real tomato dataset, which showed that introducing the pseudo-labeling method improved the fruit detection performance.

### Pseudo-label self-learning method to reduce noise labels

There is the effect of noise in the acquired pseudo labels, i.e., incorrect labeling information in the generated pseudo labels affects the training of the fruit detection model. In this paper, pseudo-label noise filtering and cycle update methods are proposed to reduce the impact of noisy pseudo labels. From Tables 4, 5, it is obvious that, as the set confidence threshold increases, the mAP value of the fruit detection model *M*_2_ increases and decreases thereupon, mainly due to the effect of the confidence threshold on the quality and quantity of the generated pseudo labels. In the real apple dataset, when the confidence threshold was 0.7, the model mAP value reached 87.5% (as show in Table 4), which is 2.3% higher than the best mAP value in Table 2. In the real tomato dataset, when the confidence threshold was 0.6, the model mAP value reached 76.9% (as show in Table 5), which is 1.7% higher than the best mAP value in Table 5.

**Table 4:** Label conversion of orange dataset to apple dataset: for the pseudo label obtained with different confidence thresholds, the pseudo-label self-learning method is further adopted to reduce the influence of noise in the pseudo label and generate a real apple dataset ${\boldsymbol{D}}_{{\boldsymbol{T}}\_{\boldsymbol{apple}}}^{\mathbf{L}}$ with higher quality labels

Model	Pseudo label	Conf	Precision	Recall	F1 Score	mAP
Improved-Yolov3	**√**	0.1	0.698	0.733	0.715	0.77
	**√**	0.2	0.747	0.749	0.748	0.79
	**√**	0.3	0.765	0.771	0.768	0.807
	**√**	0.4	0.786	0.779	0.782	0.822
	**√**	0.5	0.793	0.802	0.797	0.828
	**√**	0.6	0.801	0.796	0.798	0.847
	**√**	**0.7**	**0.828**	**0.836**	**0.832**	**0.875**
	**√**	0.8	0.814	0.808	0.811	0.847
	**√**	0.9	0.793	0.801	0.797	0.838

**Table 5:** Label conversion of orange dataset to tomato dataset: For the pseudo label obtained with different confidence thresholds, the pseudo-label self-learning method is further adapted to reduce the influence of noise in the pseudo label and generate a real tomato dataset ${\boldsymbol{D}}_{{\boldsymbol{T}}\_{\boldsymbol{tomato}}}^{\mathbf{L}}$ with higher quality labels

Model	Pseudo label	Conf	Precision	Recall	F1 Score	mAP
Improved-Yolov3	**√**	0.1	0.748	0.748	0.748	0.725
	**√**	0.2	0.757	0.751	0.751	0.731
	**√**	0.3	0.744	0.749	0.746	0.741
	**√**	0.4	0.759	0.757	0.758	0.744
	**√**	0.5	0.766	0.765	0.765	0.764
	**√**	**0.6**	**0.769**	**0.767**	**0.768**	**0.769**
	**√**	0.7	0.758	0.757	0.758	0.752
	**√**	0.8	0.743	0.747	0.745	0.748
	**√**	0.9	0.731	0.735	0.735	0.717

### Generated datasets labels

**Fig. 5:**
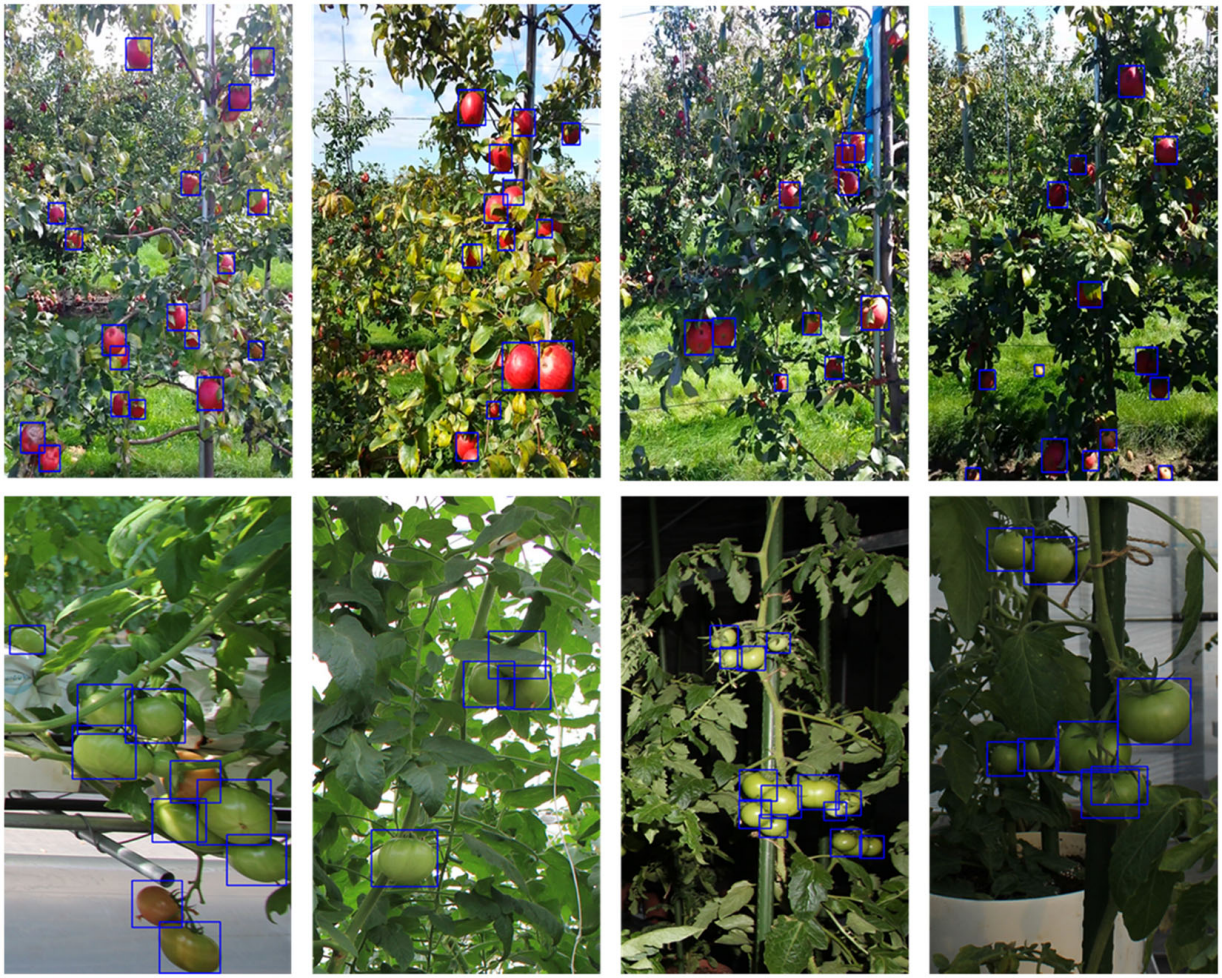
Examples of detection results of apples and tomatoes in real scenarios. The image includes target fruit in different scenarios, where the blue boxes indicate the model detection boxes, and finally, the detection boxes can be used as a ground truth for the unlabeled fruit dataset, enabling the automatic labeling of the dataset

From the comparison of the above experimental results, it is clear that the proposed method can generate higher quality label data automatically. In the real apple dataset, the mAP value of the training model reached 87.5% when obtained pseudo-labels with a confidence threshold of 0.7. In the real tomato dataset, the mAP value of the training model reached 76.9% when obtained pseudo-labels with a confidence threshold of 0.6. The above two models have also been applied to visualize apple and tomato detection in real scenarios. As shown in Fig. 5, the image includes target fruit (including apple and tomato) in various scenarios, including complex situations, such as occlusion, shadowing, and underexposure, with the blue box representing the detection results of models. In particular, most of the target fruit in the image can be detected, and the generated detection boxes can well surround the target apples at different locations in the image, which improves the quality of the generated labels, verifies the effectiveness of the proposed method in this study.

## Discussion

This paper proposed a new solution to overcome the current problem of high labeling cost for training data acquisition: the automatic labeling of unlabeled fruit datasets. The proposed method could convert labeling between labeled source fruit datasets and unlabeled target fruit datasets to achieve the automatic labeling of target fruit datasets; furthermore, it could be applied for the automatic labeling of other fruit datasets to improve the efficiency of fruit detection work in orchard.

More images of fruit species are currently available in public resources; hence, it is easier to obtain images related to the target fruit species. As shown in Table 6, we collect a large public dataset that included information on access sources, fruit species, and download addresses. It could provide a great deal of data support for subsequent experiments and facilitate experimental testing by other researchers. Therefore, by using the method in this paper, the automatic labeling of other datasets could be completed with solely a small amount of labeling information, thereby saving a great deal of data labeling work and improving fruit inspection efficiency.

**Table 6:** Information on some of the current public datasets, including the source of the dataset, the species of fruit, and the associated download URL

Source	Fruit species	Web site
Sa I^32^	Apple, Avocado, Capsicum, Mango, Orange, Rockmelon, Strawberry	http://goo.gl/9LmmOU
Bargoti S^33^	Almonds, Apple, Mango	https://data.acfr.usyd.edu.au/ag/treecrops/2016-multifruit
Koirala,A^35^	Mango	http://hdl.cqu.edu.au/10018/1261224
Kestur,R^36^	Mango	https://github.com/avadesh02
Liang Q^45^	Mango, Almond	https://pan.baidu.com/s/1pdTyVq9PlbhkR2k4Tl5zA
Hani^37^	Apple	http://rsn.cs.umn.edu/index.php/MinneApple
Tsironis V^46^	Tomato	https://github.com/up2metric/tomatOD
Laboroai	Tomato	https://github.com/laboroai/LaboroTomato
Kaggle	Tomato	https://www.kaggle.com/andrewmvd

In addition, in the practical application of this method, there are certain requirements for the source fruit and target fruit species in the fruit image transformation application: (1) the differences in shape and size between the two fruit species should be as small as possible; and (2) for the source fruit image, the background color features and the fruit color features should be distinguished as clearly as possible. Moreover, in the experimental process, the pseudo labels are mainly obtained by setting the confidence threshold manually, which has the contingency of missing the best confidence threshold. Therefore, more in-depth research on these methods is needed to solve relevant problems, so that the automatic data labeling method could be more effective in a practical level.

## Conclusion

This paper proposed a domain adaptation method for filling the species gap in deep learning–based fruit detection, which can be applied for the acquisition of labeling information from unlabeled target fruit datasets; this is a new method to solve the high data labeling cost problem. The acceptable accuracy of fruit detection by models trained on the automatically obtained labeled target fruit image showed the effectiveness of the proposed method. With this automatic labeling method, if there is solely one source fruit dataset with label, the automatic labeling of data from unlabeled target fruit dataset could be realized, saving a large amount of data labeling work. In the future, this method could be applied for the automatic labeling of more fruit datasets to improve the efficiency of orchard work.

It is worth mentioning that there is enormous scope for future research. Notably, we intend to study further on the following aspects: 1) Concerning the image transformation method used in this paper, when the fruit color features and background color features in the source fruit image are similar, the image transformation task is prone to fail. If we successfully solved the transformation problem, the method would be applicable to a wider range of fruit dataset; for this reason, how to solve the image transformation problem captures our interest. 2) During the experiments, pseudo labels are acquired by setting the confidence thresholds manually and are prone to miss the optimal threshold acquisition; hence, we plan to investigate further to obtain the best confidence threshold.

## Data Availability

Computer program codes and image data used in this study can be accessed through: https://www.github.com/I3-Laboratory/EasyDAM.

## Code availability

Computer program codes and image data used in this study can be accessed through: https://www.github.com/I3-Laboratory/EasyDAM.
